# Metabolic turnover rate, digestive enzyme activities, and bacterial communities in the white shrimp *Litopenaeus vannamei* under compensatory growth

**DOI:** 10.7717/peerj.14747

**Published:** 2023-02-15

**Authors:** Jorge Giovanni Quintino-Rivera, Regina Elizondo-González, Julián Gamboa-Delgado, Laura Teresa Guzmán-Villanueva, Alberto Peña-Rodriguez

**Affiliations:** 1 Centro de Investigaciones Biológicas del Noroeste (CIBNOR), La Paz, Baja California Sur, Mexico; 2 Conacyt–CIBNOR, La Paz, Baja California Sur, Mexico; 3 Facultad de Ciencias Biológicas, Universidad Autónoma de Nuevo León (UANL), San Nicolás de los Garza, Nuevo León, Mexico

**Keywords:** Compensatory growth, Shrimp feeding, Digestive enzymes, Metabolic turnover, Bacterial community

## Abstract

The present work aimed to evaluate the effects promoted by a phase of compensatory growth on metabolic turnover rate, digestive enzyme activity, and bacterial biota of the Pacific white shrimp *Litopenaeus vannamei* kept under different feeding regimes. Three treatments were evaluated as follows: 70% feed restriction during 3 (T3) and 6 (T6) days, followed by a period of feeding to satiety, and a control treatment without restriction periods. The results showed a full compensatory growth in treatments T3 and T6 by day 35 of the bioassay. A significant increase in trypsin and lipase (T6) activities was observed during compensatory growth, whereas specific amylase activity was significantly lower in treatment T6 compared to T3 but not significantly different from the control group. To determine the metabolic turnover rate of nitrogen in muscle tissue, an analysis of nitrogen isotope values (δ^15^N) at natural abundance levels was performed. At the end of the experimental period, shrimp under feed restriction had lower metabolic turnover rates and longer nitrogen residence times (*t*_50_) in muscle tissue, as compared to individuals in the control treatment. Regarding the changes in the bacterial communities in shrimp gut, no significant differences were observed at the phylum level, with *Proteobacteria* being the most abundant bacteria, followed by *Actinobacteria*. At family taxa level, *Rhodobacteraceae* presented the highest relative abundance in all treatments, whereas a decrease in *Vibrionaceae* was observed in treatments T3 and T6 when compared to control shrimps during compensatory growth. At the genus level, a decrease in *Celeribacter*, *Catenococcus*, and *Epibacterium*, and an increase in *Ruegeria* and *Shimia*, were identified in shrimp subjected to feed restriction when compared to control organisms during compensatory growth (day 14). At the end of the experimental period, the evaluated parameters showed similar results as those observed in the control treatment, suggesting a normalization of the metabolism and the physiological state. The present findings contribute to a better understanding on the physiological effects produced during compensatory growth in shrimp, which in turn could assist in the development of improved feeding strategies in benefit of the aquaculture industry.

## Introduction

Crustaceans represent 12.3% of the total world aquaculture production, in which the white shrimp *Litopenaeus vannamei* contributed with more than 5 million metric tons in 2019 (FAO, 2021). One of the main challenges for the expansion of shrimp culture is securing the supply of balanced feeds, which in turn can represent more than 50% of the production costs (Cummins et al., 2013; Jatobá et al., 2014). In this regard, different strategies of feed management have been proposed to reduce the production costs, including the use of trays (Casillas-Hernández et al., 2007), variations on feed frequency (Pontes, De Lima & Arruda, 2008; Ullman, Rhodes & Davis, 2019), and temporary feed restriction (Py, Elizondo-González & Peña-Rodríguez, 2022). In this regard, temporary feed restriction may promote an increased growth rate when optimal feeding conditions are restored (including decapod crustaceans); this biological response has been called compensatory growth. The compensatory response after refeeding is linked to the duration and severity of the previous feed restriction, and the length time of the refeeding period. The physiological response can lead to full, partial, or overcompensation of growth; nevertheless, if the restriction is very severe, the organism can reach a point of no return, characterized by an absence of growth compensation (Ali, Nicieza & Wootton, 2003). In this respect, Jobling (2010) describes that a recovery growth is a convergence between the compensatory growth and a catch-up growth. Moreover, feed restriction combined with other factors such as stocking density or temperature can also affect the compensatory response (Stumpf et al., 2019; Liu et al., 2022; Prates et al., 2023).

In farmed shrimp, feed restriction has been evaluated at different degrees of restriction and time periods, where shrimp has showed full compensatory growth with a concomitant significant reduction on feed utilization (Maciel, Francisco & Miranda-Filho, 2018; Liu et al., 2022), and in other cases with partial growth recovery (Yildirim & Aktaş, 2019; Rocha et al., 2019). Furthermore, other benefit of the use of this strategy is the reduction in nitrogen and phosphorous residual compounds in the farm effluents and environment (Zhu et al., 2016; Lucas, Southgate & Tucker, 2019). On the other hand, during feed restriction, the decreased nutrient intake can alter the metabolic rate of organisms, forcing them to recycle the available nutrients for their elementary physiological processes, thus limiting their growth (Gamboa-Delgado et al., 2011). In crustaceans, a decrease in digestive enzymes activities is often observed during starvation, affecting the energy supply to the organism (Charron et al., 2014; Sacristán et al., 2016). In this regard, the energetic compounds can be differentially mobilized (Sacristán et al., 2017; Calvo et al., 2018) and the preference to obtain energy can also be modified, as shown in *L. vannamei* shrimp, in which glucose, glycogen, and lipids in the hepatopancreas decrease after a short period of starvation (Sánchez-Paz et al., 2007). Nevertheless, some other studies have not reported negative effects on digestive enzymes activities after cyclic periods of feeding and fasting, as observed in *Cherax quadricarinatus* juveniles (Stumpf, Tropea & Greco, 2014). Moreover, feed restriction strategies can affect the bacterial community structure in the digestive tract of organisms (Sacristán et al., 2016; Eslamloo et al., 2017), which could, in turn, have an impact on the immune response of the host, by increasing the risk of diseases caused by pathogenic bacteria (Brown et al., 2012; Foysal et al., 2020).

During compensatory growth, and after returning to favorable feeding conditions, new physiological adaptations occur, and these include metabolic adjustments promoting elevated growth efficiencies, as described for different farmed fish and crustacean species (Py, Elizondo-González & Peña-Rodríguez, 2022). The increase of feed intake allows the reactivation of digestive enzymatic activities that enhance digestion and absorption of nutrients (Rocha et al., 2019; Zaefarian et al., 2020). This adaptation usually reflects hyperphagia and improvement of feed conversion efficiency, revealing a restoration of energy reserves used during feed restriction (Furné et al., 2012; Won & Borski, 2013). At the microbiome level, some studies in fish have described changes in the bacterial digestive structure after re-feeding, suggesting a beneficial relationship with physiological processes associated to metabolism and immune system (Liu et al., 2020; Sakyi et al., 2020).

The promotion of compensatory growth to reduce operating costs during animal production is a well-documented strategy (Py, Elizondo-González & Peña-Rodríguez, 2022); nevertheless, there is still relevant information to disclose on the physiological effects occurring in the white shrimp *L. vannamei*. In this context, the present study aims to provide new insights on the physiological responses occurring during compensatory growth, by evaluating the metabolic turnover rate of nitrogen in muscle, by measuring the digestive enzyme activities and changes in the bacterial communities of the digestive tract of juvenile *L. vannamei*.

## Materials and Methods

### Experimental feeds and shrimp pre-conditioning

Two experimental diets were formulated: (a) a pre-conditioning diet and (b) a reference diet (Table 1). The pre-conditioning diet was manufactured with poultry by-product meal (55%) to promote a specific isotopic signature in shrimp before the feed restriction assay. The reference diet was based on fish meal as main ingredient (56%) having a different isotopic signature for nitrogen. In this way, the dietary shift had the objective of promoting clear, exponential isotopic changes that eventually allowed estimating the metabolic turnover rate of nitrogen. The formulation and manufacture of the experimental diets was done as described in Peña-Rodríguez et al. (2020), using a 1.5 L mixer and a meat grinder to produce pellets through a 2-mm die. The chemical proximal composition of the experimental diets was analyzed according to AOAC (2005) methods for moisture (Method 930.15), lipids (Method 2003.05), crude fiber (Method 978.10), ash (Method 942.05), and crude protein by Dumas method (Ebeling, 1968).

**Table 1:** Ingredient composition (g kg^−1^ diet) and proximate analysis (% as is) of experimental diets.

	Experimental diets	
Ingredients	Pre-conditioning	Reference
Fish meal^a^	160	560
Poultry by-product meal^b^	550	0
Corn starch^c^	254	344
Fish oil^a^	20	10
Soy lecithin^d^	0	70
Alginic acid^c^	10	10
Vitamin-mineral premix^f^	5	5
Vitamin C^g^	1	1
Proximate composition		
Moisture	9.2 ± 0.07	9.3 ± 0.01
Protein	36.8 ± 0.08	35.6 ± 0.07
Lipids	9.2 ± 0.04	9.7 ± 0.05
Crude fiber	0.23 ± 0.01	0.12 ± 0.01
Ash	14.5 ± 0.04	12.4 ± 0.07
NFE^h^	30.01	32.85

**Notes:**

a PMA S.A. de C.V., Jalisco, MX.

b CYGA de México, CDMX, MX.

c Sigma Aldrich, St. Louis, MO, US.

d Suministros AZ, Baja California Sur, MX.

e Sigma Aldrich, St. Louis, MO, US.

f Vitamin-mineral premix: detailed content in Peña-Rodríguez et al. (2020)

g Prilabsa, Sinaloa, MX.

h Nitrogen-free extract (estimated by subtracting the percentages of crude protein, lipids, crude fiber, and ash from 100%).

Pacific white shrimp *L. vannamei* having an average weight of 45 ± 8 mg were donated by a commercial facility (Larvas Gran Mar, SA de CV, Baja California Sur, MX). Shrimp were received at the Aquaculture Nutrition Laboratory at CIBNOR, and acclimated for 30 days in a 800-L fiber glass tank under controlled conditions (28.1 ± 0.4 °C, pH 8.1 ± 0.2, D.O. > 4.5 mg L^−1^, photoperiod of 12 h:12 h light:dark). The system had 60% daily water exchange and marine water (37 UPS) was treated through a 1-μm mesh and sterilized with UV light. During the acclimation period, shrimp were fed to satiety twice a day (09:00 and 15:00 h) with the pre-conditioning diet, formulated to confer a known isotopic baseline in shrimp muscle tissue before the compensatory growth experiment.

### Experimental design of the compensatory growth trial

After the acclimation period, three different dietary treatments were evaluated for 35 days: (a) Control treatment: feed to satiety, (b) T3: 3 days of 70% feed restriction with respect to control group, and (c) T6: 6 days of 70% feed restriction with respect to control group. Four replicates per treatment were placed in day 1, and at day 28 after sampling, shrimps in the 4^th^ replicate of each treatment were homogeneously redistributed to the rest of replicates to maintain similar shrimp density as in the beginning of the experiment. All shrimp were fed with the reference diet. For each replicate, a fiber glass tank of 60-L capacity was used to hold 12 shrimp (440 ± 30 mg, initial avg. wt.) randomly distributed in each tank. The Control group was initially fed at 7% of the biomass, and after the second experimental day, feeding was adjusted according to observed feed consumption. In the case of T3 and T6, shrimp were fed to satiety after the restriction period. All groups were fed their respective feed amount divided in two rations (09:00 h and 15:00 h). Every day (08:00 h) all the experimental tanks underwent a 60% water exchange, removing feed residues, molts and feces by siphoning. During the experimental period, shrimp performance was estimated in terms of final weight (FW), weight gain (WG (%) = 100 × (FW − Initial weight)/Initial weight), specific growth rate (SGR (% day^−1^) = 100 × (ln FW − ln Initial weight)/days), feed intake (FI (g) = Total of feed provided per shrimp during the feeding trial), feed conversion ratio (FCR = FI/(FW − Initial weight)) and survival (S (%) = final number of shrimp/initial number shrimp without considering those sampled × 100).

In order to obtain an indirect estimation of the metabolic turnover rate of nitrogen in muscle tissue, stable isotope analysis was applied to samples collected at different times (Gamboa-Delgado et al., 2020). During the acclimation period, shrimp received a diet having a known nitrogen isotope value and this diet established an isotopic baseline in shrimp before feeding treatments were applied. It was expected that the dietary shift would elicit exponential isotopic changes, to enable the ensuing metabolic calculations. At experimental day 0, six shrimp were sampled and afterwards three shrimp per treatment were randomly sampled on experimental days 3, 6, 9, 14, 21, 28, and 35. Shrimp were euthanized in an ice/water slurry, and the abdominal muscle was dissected and kept at −80 °C until pretreatment for isotopic analysis. In the case of digestive enzymes activity, the hepatopancreas of four shrimp per treatment was sampled at days 9, 14, and 35, and preserved at −80 °C until use. For the high-throughput 16S rRNA gene analysis applied to evaluate bacterial communities, the digestive tract (stomach and intestine) of four shrimp per treatment were aseptically sampled at days 14 and 35, then kept in vials with 90% ethanol at −20 °C until DNA extraction.

### Stable isotope analysis of shrimp muscle

Muscle tissue samples from shrimp under different treatments were desiccated at 55 °C until constant weight and ground in a mortar until a fine homogenous powder was obtained (Gamboa-Delgado et al., 2011). For elemental and isotopic analyses (δ^15^N), between 900 and 1,100 µg of each sample were packed in tin microcapsules (041077; Costech Analytical Technologies Inc, Valencia, CA, USA), and sent to the Stable Isotope Facility (University of California-Davis, Davis, USA). Samples were analyzed in a PDZ Europa ANCA-GSL elemental analyzer interfaced to a PDZ Europa 20-20 isotope ratio mass spectrometer (Sercon Ltd., Cheshire, UK). Isotopic results are expressed in delta notation (δ), which is defined as parts per thousand (‰) deviations from the δ^15^N values of the international standard (atmospheric nitrogen).

For the estimation of nitrogen turnover rate and residence half-time (*i.e*., the time period necessary to replace 50% of the muscle nitrogen with the “new” dietary nitrogen, *t*_50_), isotopic changes in shrimp muscle were monitored throughout the experimental period after the dietary switch from the pre-conditioning feed to the feed supplied after treatments were diversified. The metabolic nitrogen turnover rate (*m*) in shrimp muscle tissue was estimated by incorporating the isotopic values of nitrogen (δ^15^N), and the variable time, into an exponential model describing isotopic change (*C = C*_*n*_ *+ (C*_*0* −_ *C*_*n*_*)e*^*−(k+m)t*^) (Hesslein, Hallard & Ramlal, 1993). Values of *k* were obtained by fitting an exponential growth model to observed weights, *k* = *log* (final weight/initial weight)/time (days). The coefficients of this model indicate the relative magnitude of isotopic change in relation to growth (*k*) and metabolic turnover (*m*). Parameter *m* is unknown and it was estimated using iterative non-linear regression. In the equation, C_0_ is the isotopic value at the beginning of the experimental period, *C*_*n*_ represents the isotopic value of the consuming animal at equilibrium with the new feed, and *C* the isotopic value at time *t*. The *k* and *m* coefficients were used to estimate the nitrogen residence time in muscle tissue (*t*_50_ = *In2*/*m* + *k*) (MacAvoy, Macko & Arneson, 2005). The estimated values were based on the growth observed after the feeding restriction periods.

### Digestive enzyme activities

To determine the digestive enzyme activities, the hepatopancreas of shrimps sampled from their respective treatments, were individually weighed and homogenized in distilled water (1:4 w/v ratio) using a Fastprep-24 homogenizer (MP Biomedicals, Irvine, CA, USA). Then, samples were centrifuged at 15,000 *g* for 15 min at 4 °C (Schleder et al., 2018). The supernatant was recovered and stored at −80 °C until the quantification of enzymatic activities using a multimode microplate reader Varioskan Flash (Thermo Fisher Scientific, Waltham, MA, USA).

Total protein was quantified according to Bradford (1976), at 595 nm absorbance, using bovine albumin as standard. The digestive enzyme activities in the shrimp hepatopancreas were expressed in units per milligram of protein (U mg protein^−1^). Trypsin activity was evaluated according to Toledo-Cuevas et al. (2011) using Boc-Gln-Ala-Arg-7-amido-4-methylcoumarin hydrochloride as substrate (B4153; Sigma-Aldrich, St. Louis, MO, USA). The activity was read at 460 nm (excited at 355 nm) each 30 s for 30 min. Lipase was evaluated according to Gilannejad et al. (2020), using 4-methylumbelliferyl butyrate as substrate (19362; Sigma-Aldrich, St. Louis, MO, USA). The fluorescence was measured at 450/365 nm (emission/excitation). Amylase activity was determined spectrophotometrically at 570 nm by the method described by Vega-Villasante, Nolasco & Civera (1993) using starch as a substrate. All activities were evaluated per quadruplicate and, during handling, samples were kept in ice to prevent enzyme degradation.

### 16s rRNA gene analysis

The DNA was extracted from the shrimp digestive tracts using a DNeasy Blood & Tissue kit (Qiagen, Hilden, Germany). The DNA’s quality and quantity were determined by electrophoresis in agarose gels and with a Qubit® 3.0 fluorometer (Thermo Fisher Scientific, Waltham, MA, US), respectively. The hypervariable regions V3 and V4 of the 16S rRNA gene on each sample (*n* = 4, per treatment/time) were amplified with primers proposed Klindworth et al. (2013): Forward Primer 5′ (TCGTCGGCAGCGTCAGATGTGTATAAGAGACAGCCTACGGGNGGCWGCAG) and Reverse Primer 5′ (GTCTCGTGGGCTCGGAGATGTGTATAAGAGACAGGACTACHVGGGTATCTAATCC). A second PCR was performed to attach dual indices and Illumina sequencing adapters in all PCR fragments using Nextera index kit v2 (FC-121-1011; Illumina, San Diego, CA, US). For both PCRs, the amplicons were purified using AMPure XP (A63881; Beckman Coulter, Brea, CA, US). The library was sequenced at CIBNOR using an Illumina MiSeq system and MiSeq reagent V3 (600 cycle) kit, following the manufacturer’s protocol (Illumina, San Diego, CA, USA).

Sequences were processed using MOTHUR v1.41.3 software (Schloss et al., 2009), following the workflow described by Kozich et al. (2013). Reads with quality of Q > 25, length > 250 bp, and ambiguous nucleotides were discarded. Paired-end reads were aligned to assign OTUs (phylum to genus taxa levels when possible) using the SILVA v138 database at 97% confidence (Quast et al., 2013). Alpha diversity was estimated using the Shannon diversity index and the Chao1 richness estimator. In addition, the R package DESeq2 (Love, Huber & Anders, 2014) was employed for differential abundance analysis within taxa levels. Raw sequences were deposited at the NCBI under BioProject PRJNA880846.

### Statistical analysis

A one-way ANOVA was used to compare the determined zootechnical parameters, *k*, *m*, and *t*_50_ values, and enzymatic digestive activities. Data were then analyzed by Tukey multiple comparison test, if applicable. Tests were conducted with a significant criterion set at *p* < 0.05. All statistical analyses were performed using R v1.417.

## Results

### Shrimp growth performance

**Figure 1:**
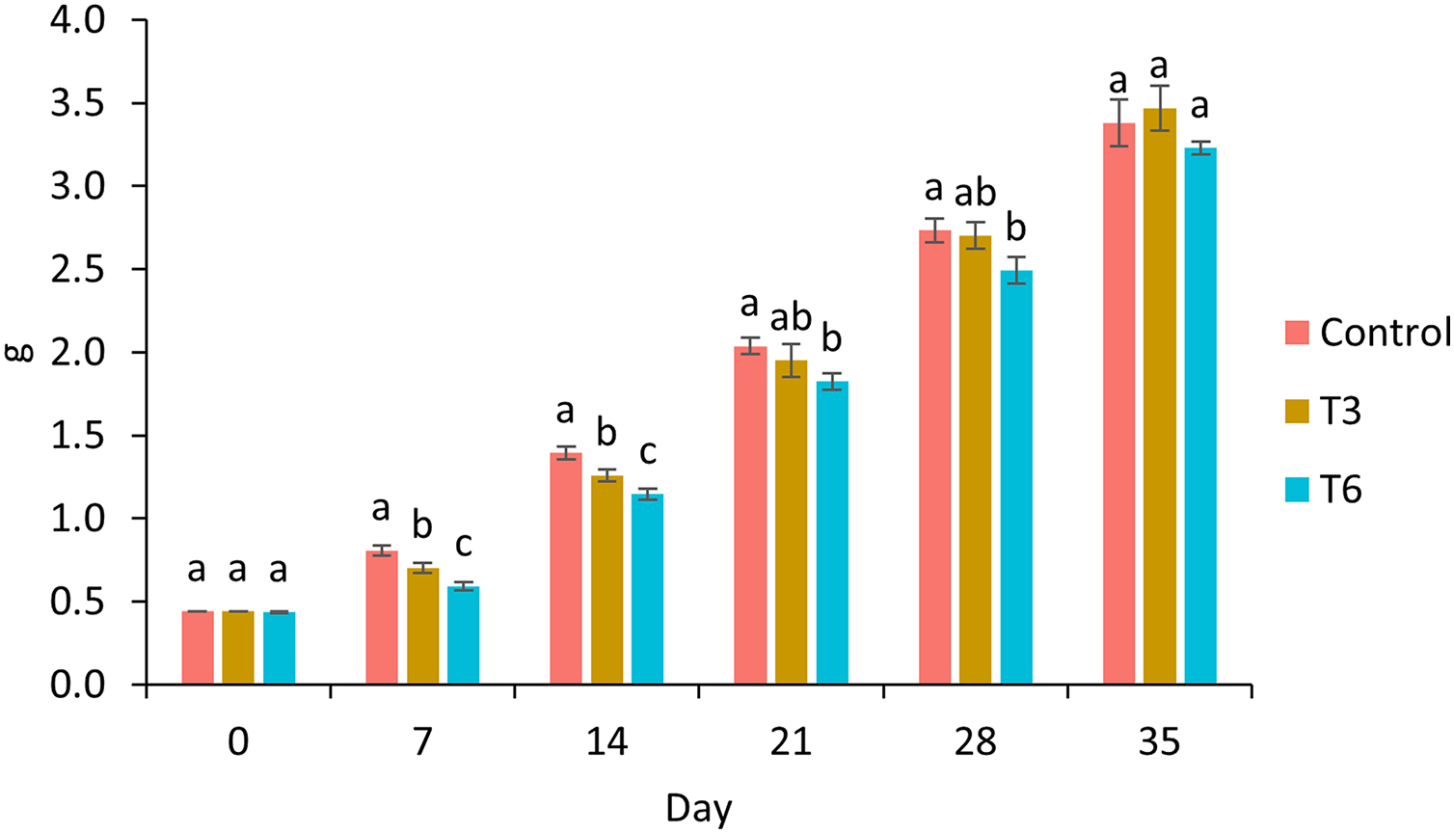
Growth of shrimp fed without restrictions (Control) and subjected to a 70% feed restriction during 3 (T3) and 6 days (T6). Different letters indicate significant difference (*p* < 0.05) among treatments in the same time period determined by Tukey’s test.

Survival of shrimp during the experimental trial was higher than 90% for all treatments. After 7 and 14 days of the experimental period, shrimp under 70% feed restriction during 3 (T3) and 6 (T6) days showed significantly lower growth compared to the control group (F_2,11_ = 198.03, *p* < 0.001 and F_2,11_ = 134.68, *p* < 0.001 respectively) (Fig. 1). In this regard, day 7 marked the end of the restriction period for shrimp under T6, meanwhile shrimp under T3 were 3 days feeding to a favorable condition. In contrast, on day 14, shrimps under regimes T6 and T3 were fed to satiety for 7 and 10 days, respectively. On day 21 and 28, a significant lower weight was observed in T6 shrimp and Control treatment (F_2,11_ = 14.56, *p* = 0.002 and F_2,11_ = 15.05, *p* = 0.001 respectively). Finally, on experimental day 35, shrimp under T3 treatment showed the highest final weight (3.47 ± 0.13 g) compared to Control (3.38 ± 0.14 g) and T6 (3.23 ± 0.04) treatments; nevertheless, no statistical differences were observed (F_2,8_ = 3.27, *p* = 0.11), as well as for weight gain and specific growth rate (SGR) parameters (*p* > 0.05) (Table 2).

**Table 2:** Shrimp *L. vannamei* performance and survival after 35 days of feeding on different regimes.

Treatments	WG (%)	SGR (% day^−1^)	FI (g)	FCR	Survival (%)
Control	668 ± 32	5.82 ± 0.12	3.91 ± 0.08^c^	1.32 ± 0.05^b^	100 ± 0
T3	676 ± 28	5.86 ± 0.10	3.70 ± 0.02^b^	1.23 ± 0.05^a^	97 ± 6
T6	640 ± 11	5.72 ± 0.04	3.44 ± 0.02^a^	1.23 ± 0.02^a^	93 ± 12

**Note:**

Mean initial weight: 0.44 ± 0.03 g. Control: shrimps without feed restriction, T3: 3 days of 70% feed restriction as compared to Control, and T6: 6 days of 70% of feed restriction as compared to Control. Values are given as mean ± SD (*n* = 3). Different letters indicate significant differences (*p* < 0.05) among treatments in the same column determined by Tukey’s test.

**Figure 2:**
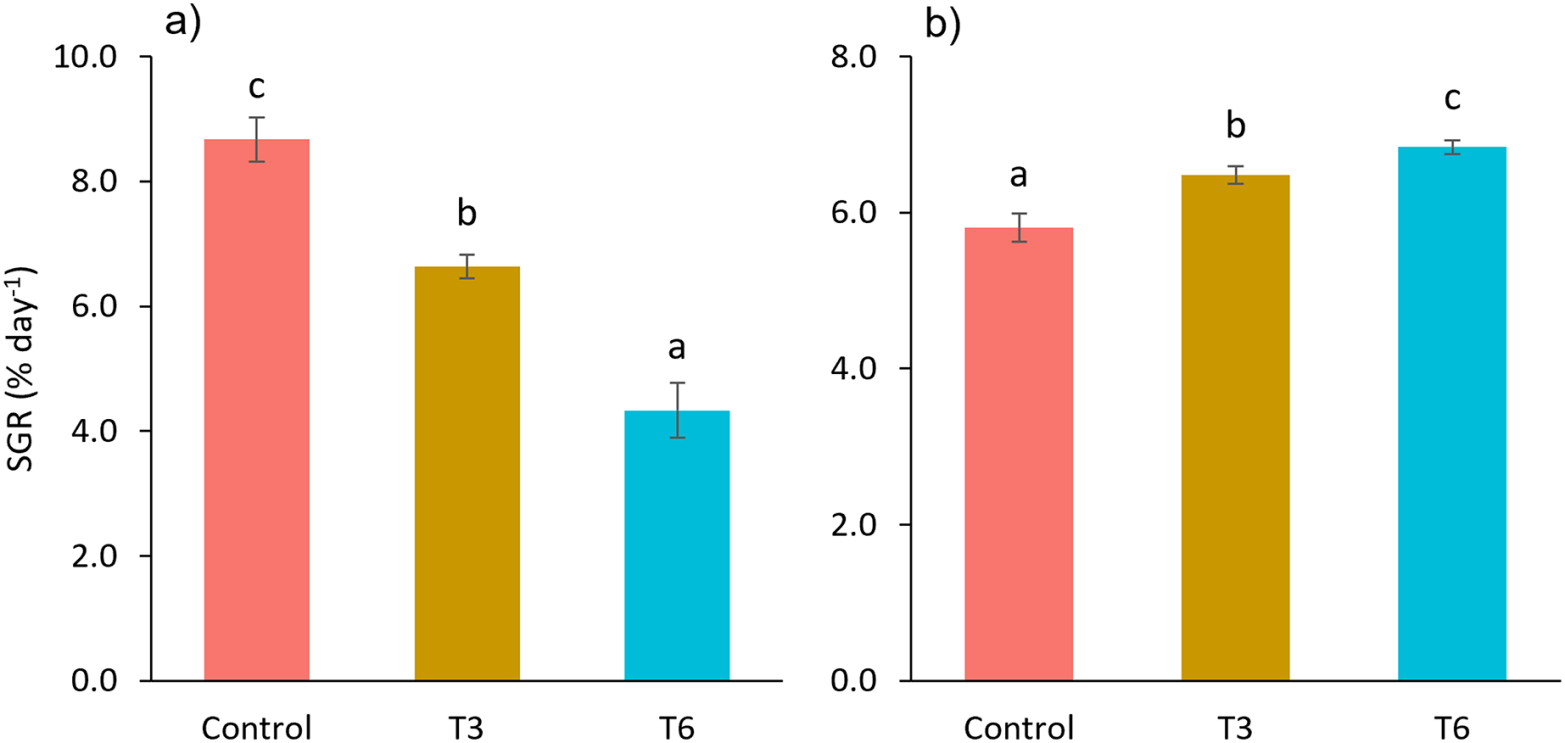
Specific growth rate (SGR, % day^−1^) from day 1 to 7 (A), and 7 to 28 (B), of shrimp *L. vannamei* fed without restrictions (Control) and subjected to a 70% feed restriction during 3 (T3) and 6 days (T6). Different letters indicate significant difference (*p* < 0.05) among treatments in the same time period determined by Tukey’s test.

**Figure 3:**
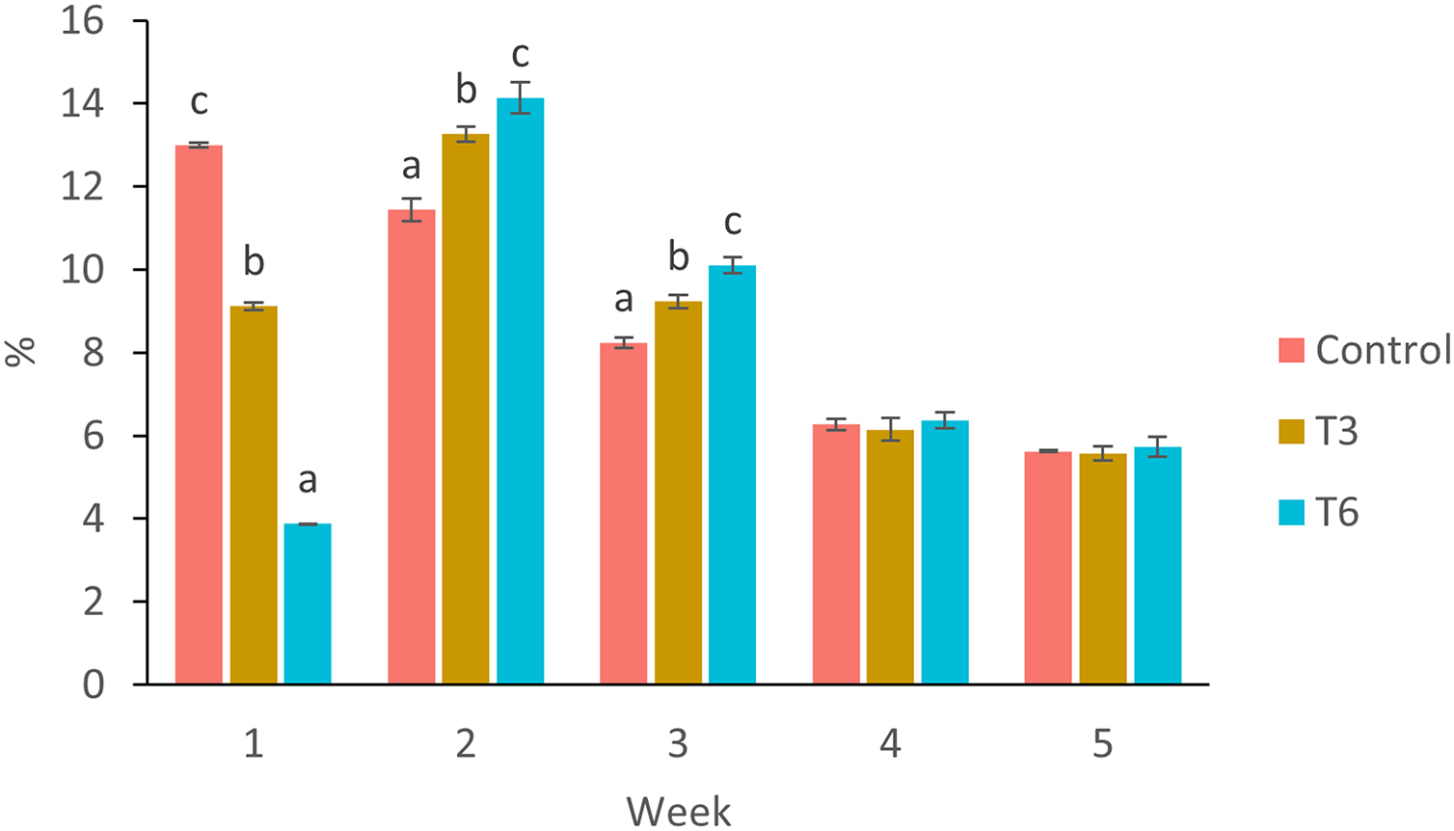
Mean weekly feeding rate according to biomass (%) of shrimp fed without restrictions (Control) and subjected to a 70% feed restriction during 3 (T3) and 6 days (T6). Different letters indicate significant difference (*p* < 0.05) among treatments in the same time period determined by Tukey’s test.

Considering the shrimp growth between days 1 and 7, the SGR was significantly higher for shrimp in the Control treatment compared to feed-restricted shrimps (F_2,8_ = 129.66, *p* < 0.001) (Fig. 2a). Nevertheless, during the recovery period without feed restrictions (from day 7 to 28), the SGR was significantly higher for T3 and T6 treatments compared to Control (F_2,8_ = 46.20, *p* < 0.001) (Fig. 2b), reflecting a compensatory growth with a full recovery of growth at day 35. Final feed intake (FI) and FCR were significantly lower for the restricted organisms when compared to Control shrimps (F_2,8_ = 72.32, *p* < 0.001 and F_2,8_ = 7.06, *p* = 0.027 respectively). In this regard, during the refeeding period, hyperphagia was evident in shrimp under the restricted treatments, specifically from the end of the restriction until day 14 (Fig. 3), where a mean daily feed consumption equivalent to 11% of shrimp biomass was observed in the control treatment, in contrast to 13% and 14% in treatments T3 and T6 respectively (F_2,9_ = 132.33, *p* < 0.001).

### Isotopic analysis and nitrogen turnover rate

**Figure 4:**
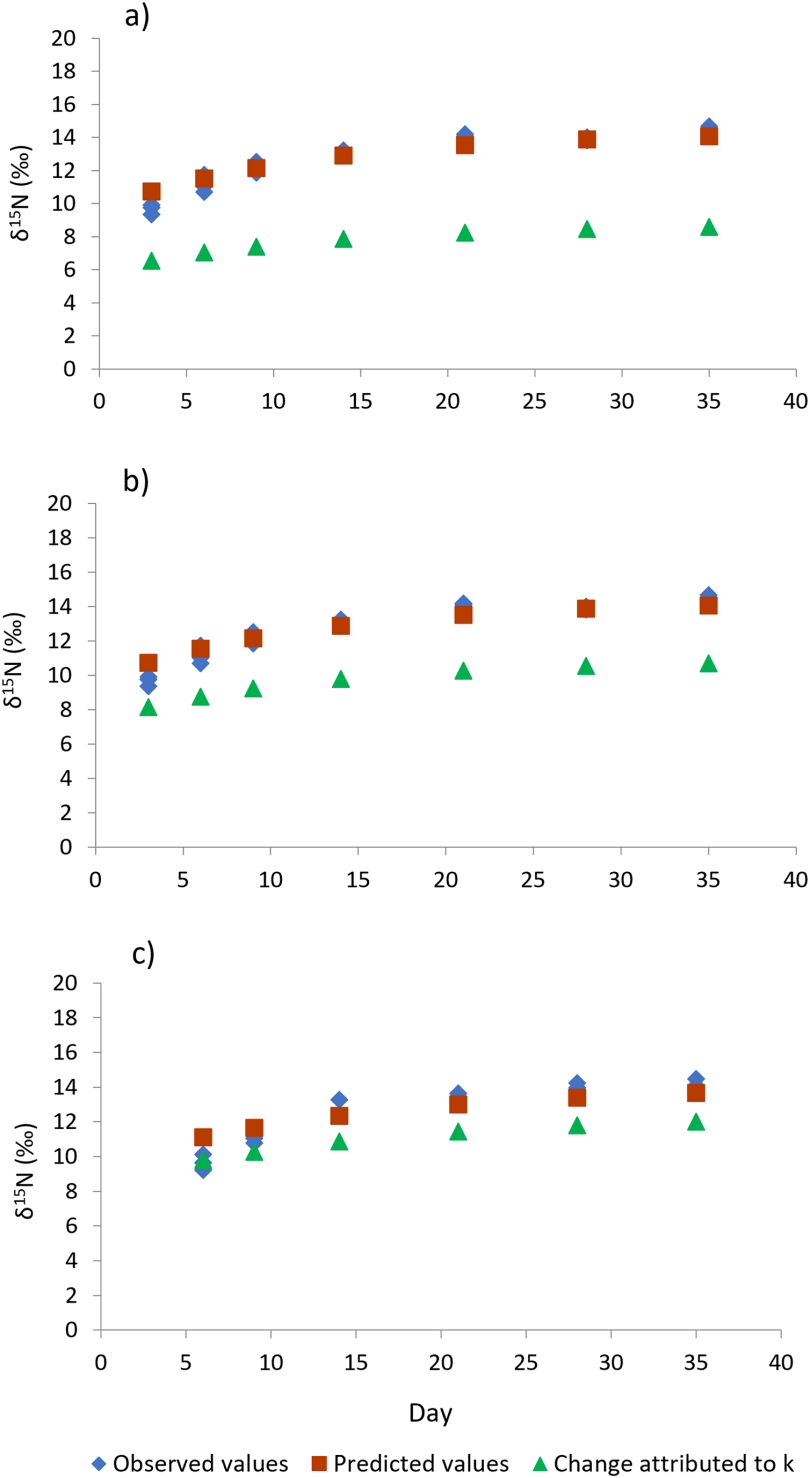
Isotopic changes in muscle tissue conferred by the diet in shrimp *L. vannamei* fed without restrictions (A) and subjected to a 70% feed restriction during 3 (B) and 6 days (C). Blue rhombi represent observed δ^15^N values over the experimental period. Orange squares represent δ^15^N predicted values indicated by the model of Hesslein, Hallard & Ramlal (1993). This equation allows estimating the relative contributions of growth and the metabolic turnover rate (*k* + *m*) to the observed isotopic change. Green triangles show the isotopic change attributed to growth only (*k*).

Shrimp muscle tissue at the beginning of the feed restriction experiment had a δ^15^N value of 8.53‰ ± 0.1‰, which was established by the pre-conditioning diet. At the end of the 35-days experiment, all treatments caused an isotopic balance between the δ^15^N values of the diet and muscle tissue. An asymptotic δ^15^N value of 14.31‰ ± 0.26‰ indicated the end of the exponential isotopic shift. The isotopic changes observed during the experimental period, and the contribution of the metabolic turnover rate (*m*) and growth rate (*k*) to the overall isotopic changes are shown in Fig. 4. Table 3 shows the estimated nitrogen turnover rate and half-time residence (F_2,8_ = 76.0, *p* < 0.001) in shrimp muscle tissue after feeding restriction (during compensatory response). Growth rate was higher for shrimp under T6 treatment, followed by T3 and finally the Control (F_2,8_ = 46.5, *p* < 0.001). Estimated nitrogen metabolic turnover rates ranged from 0.008 to 0.033 day^−1^ and values determined in shrimp under the Control treatment were higher when compared to T3 and T6 (F_2,8_ = 239.6, *p* < 0.001). The contribution of growth (tissue accretion) to the isotopic change was higher for T6 (88%) and T3 (76%) than for shrimp under the Control treatment (61%). In the same way, longer half-time residence times of nitrogen in tissue were observed in muscle tissue of animals kept under treatments T3 and T6, as compared to the Control shrimp.

**Table 3:** Growth rates (*k*), estimated nitrogen metabolic turnover rates (*m*), and half-times (*t_50_*) in muscle tissue of shrimp *L. vannamei* fed without restrictions (Control) and subjected to a 70% feed restriction during 3 (T3) and 6 days (T6). Estimations were applied from data observed from experimental day 7 to 35.

Treatments	*k* (day^−1^)	*m (day^−1^)	*k m* contribution to isotopic change (%)	*t*_50_ (days)
Control T3 T6	0.051 ± 0.0013^a^ 0.057 ± 0.0014^b^ 0.061 ± 0.0015^c^	0.033 ± 0.0012^a^ 0.018 ± 0.0017^b^ 0.008 ± 0.0015^c^	61 – 39 76 – 24 88 – 12	8.3 ± 0.11^a^ 9.2 ± 0.17^b^ 10.0 ± 0.20^c^

**Note:**

*m values were estimated using iterative non-linear regression from the equation of isotopic exponential change proposed by Hesslein, Hallard & Ramlal (1993). Different letters indicate significant difference (*p* < 0.05) among treatments in the same column determined by Tukey’s test.

### Digestive enzyme activity

**Figure 5:**
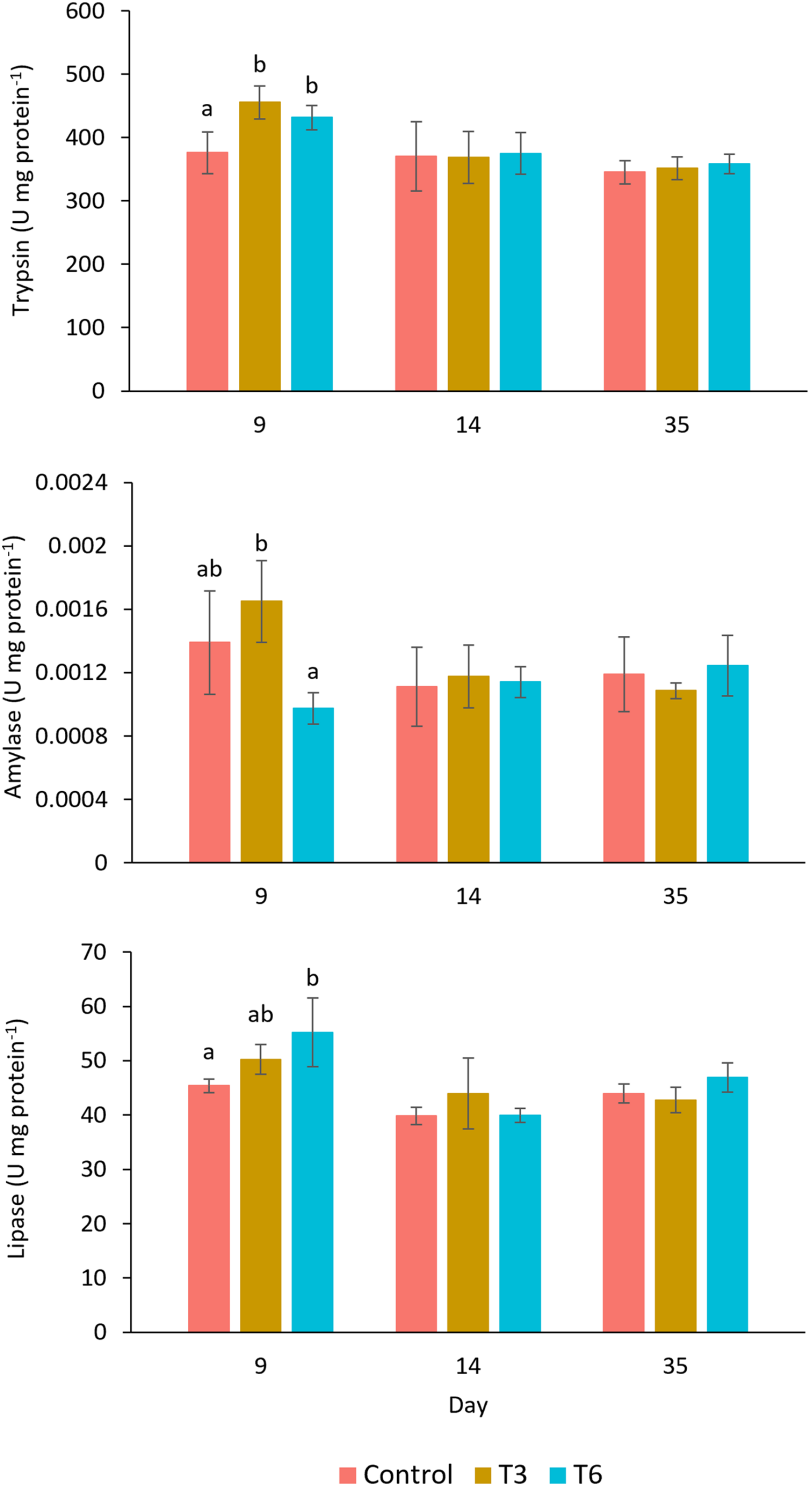
Enzymatic digestive activity of trypsin, amylase, and lipase in shrimp *L. vannamei* fed without restrictions (Control) and subjected to a 70% feed restriction during 3 (T3) and 6 days (T6) (*n* = 4). Different letters indicate significant differences (*p* < 0.05) among treatments in the same time period determined by Tukey’s test.

The digestive enzyme activities measured in the shrimp’s hepatopancreas are shown in Fig. 5. The trypsin activity at day 9, during compensatory response, showed a significantly higher activity in shrimps belonging to T3 and T6, as compared to values observed in shrimp in the Control treatment (F_2,11_ = 9.24, *p* = 0.007). Nevertheless, after day 14, no significant differences were shown among treatments (*p* > 0.05). In the case of amylase at day 9, shrimp under the T6 regime showed significantly lower activity than T3 (F_2,11_ = 7.65, *p* = 0.011), but activity values were not significantly different from the Control. Lipase activity showed higher activity in treatment T6 at day 9, as compared to the rest of treatments (F_2,11_ = 5.96, *p* = 0.023). After day 14, trypsin, amylase, and lipase activities did not show significant differences among treatments.

### Bacterial digestive biota

**Figure 6:**
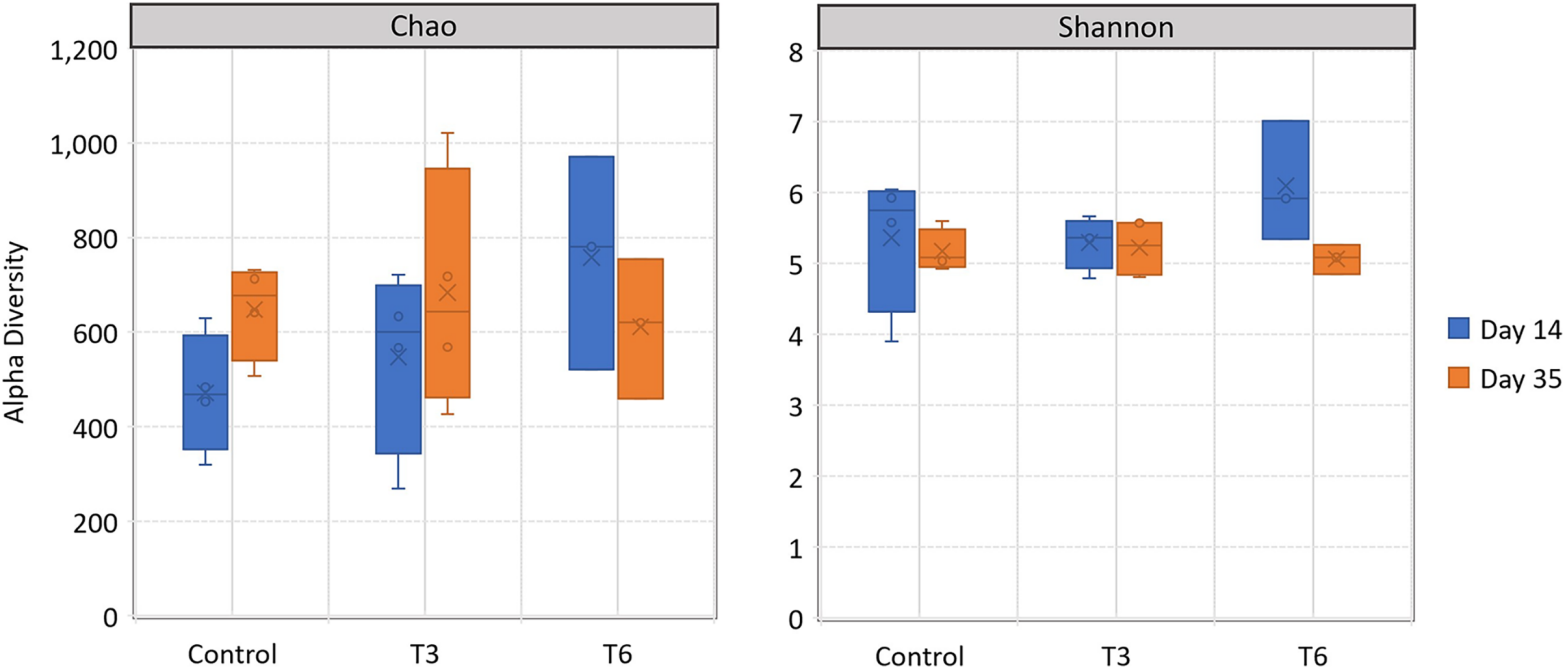
Alpha-diversity, in terms of richness by Chao estimator and evenness according to Shannon index, in the digestive tract of shrimp *L. vannamei* fed without restrictions (Control) and subjected to a 70% feed restriction during 3 (T3) and 6 days (T6).

**Figure 7:**
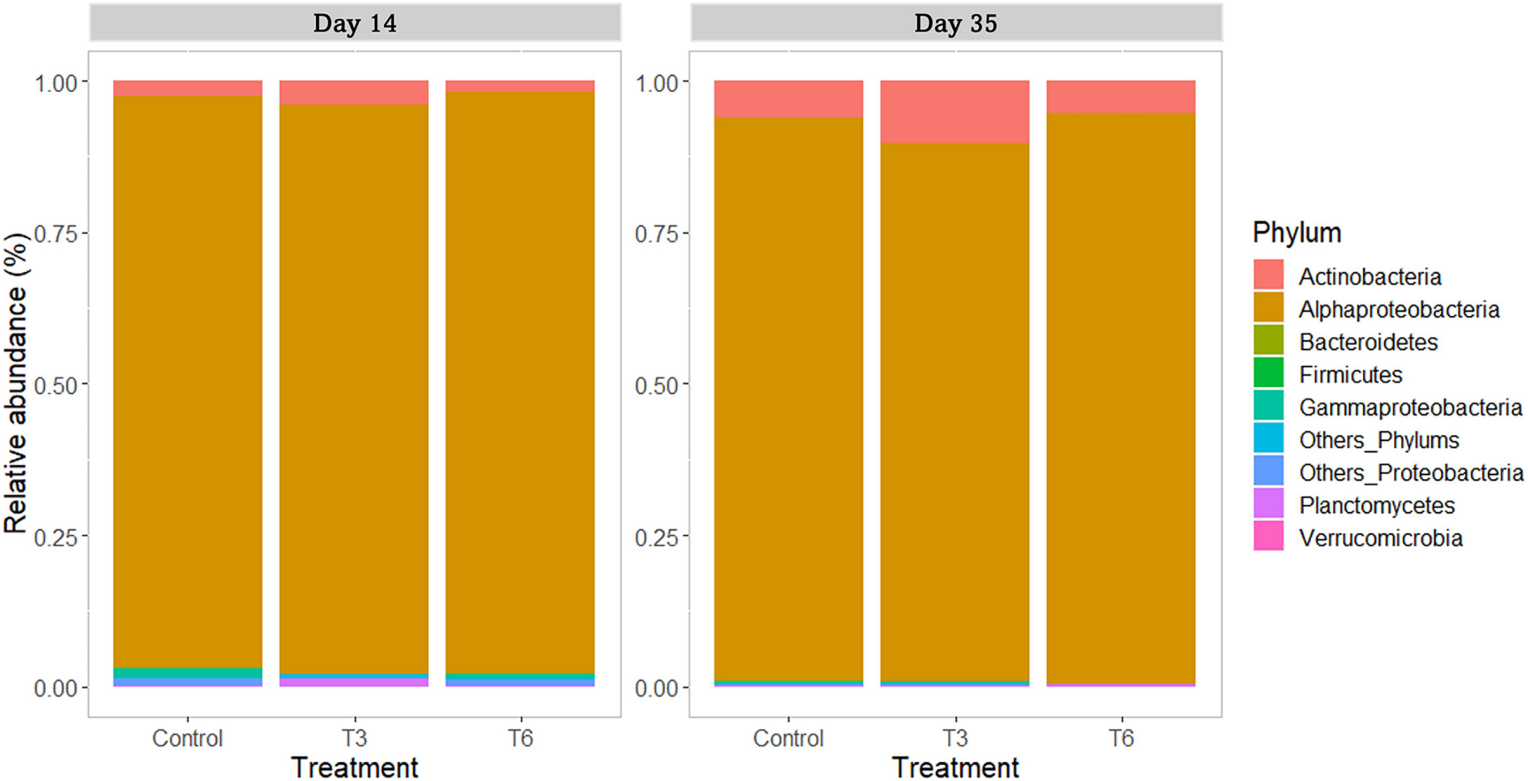
Relative abundance of main bacterial phyla in shrimp *L. vannamei* digestive tract fed without restrictions (Control) and subjected to a 70% feed restriction during 3 (T3) and 6 days (T6) (*n* = 4).

All replicate samples showed an average of 95,367 sequences assigned to 455 different OTUs. According to alpha-diversity in terms of Chao and Shannon indexes, the shrimp bacterial community in the digestive tract of shrimp were statistically similar (*p* > 0.05) among treatments and between sampling days (14 and 35) (Fig. 6). At phylum level, the digestive tract of shrimp did not show significant difference among treatments at both sampling times. A high dominance of *Proteobacteria* (>85%), followed by *Actinobacteria* (up to 10%) and reduced abundance of *Bacteriodetes* and *Firmicutes* (<2%) were observed for all samples (Fig. 7).

**Figure 8:**
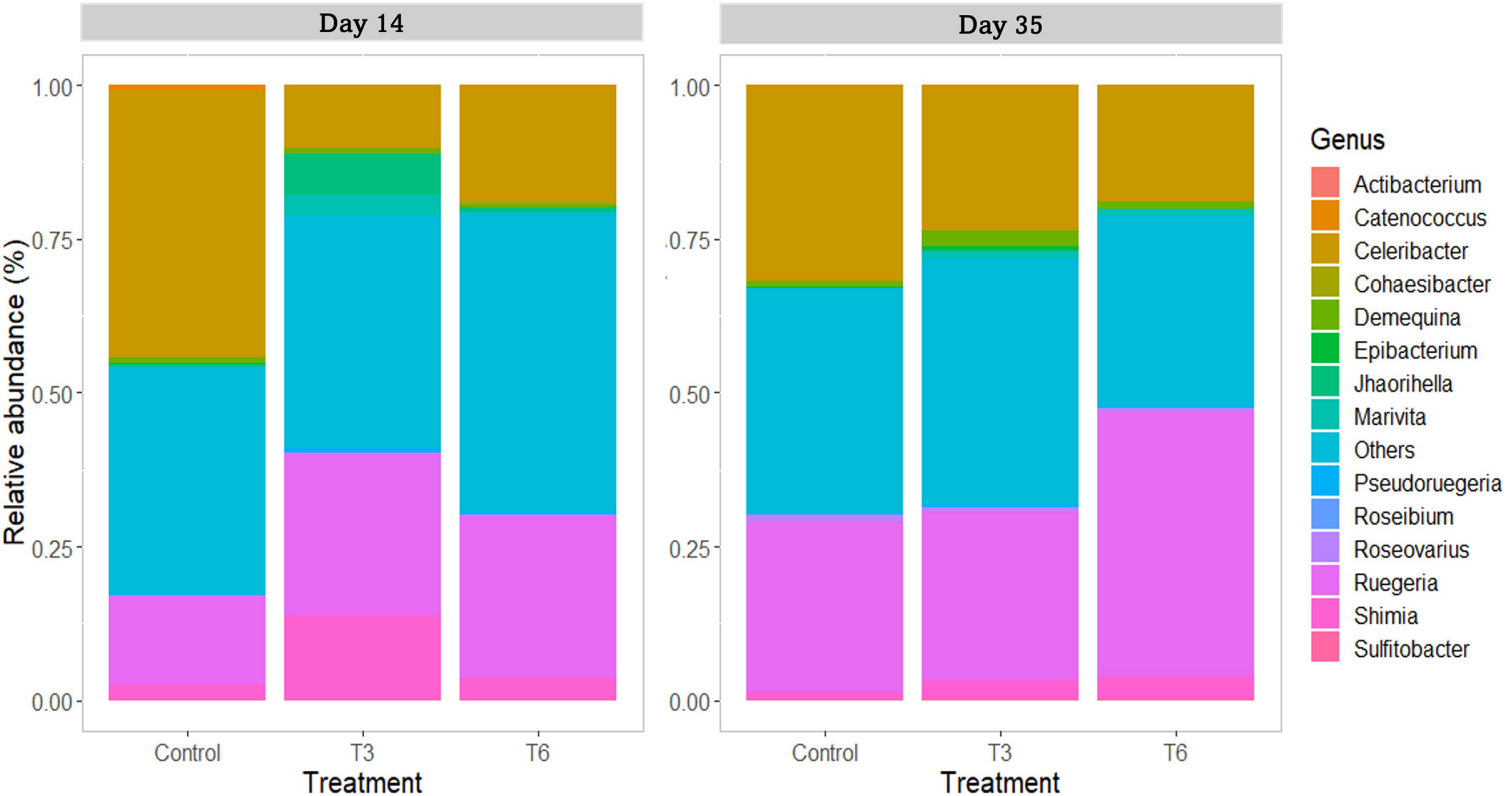
Relative abundance of main bacterial genus in shrimp *L. vannamei* digestive tract fed without restrictions (Control) and subjected to a 70% feed restriction during 3 (T3) and 6 days (T6) (*n* = 4).

For the family taxa level, *Rhodobacteraceae* was the most abundant group (>80%) among treatments at days 14 and 35 (*p* > 0.05). At day 14, only *Vibrionaceae* showed a significant reduction in treatments T3 and T6 under compensatory growth (0.02–0.11%) compared to Control shrimp (1%) (*p* < 0.05). At genus level (Fig. 8), shrimp under Control treatment showed a significantly higher relative abundance of *Celeribacter* (37.6%), *Catenococcus* (0.95%), and *Epibacterium* (0.34%) at day 14, while relative abundances were lower in treatments T3 and T6 (≤19%, ≤0.11%, and <0.1%, respectively) under compensatory growth. In the same period, the *Cohaesibacter* genus was observed in higher relative abundance in T6 treatment than in Control shrimp. On the other hand, *Ruegeria* represented one of the most abundant genus among treatments, having higher relative abundances in T3 and T6 (28.6% and 26.2%, respectively) as compared to control group (16.2%). In the same way, the *Shimia* genus was also more relatively abundant in T3 and T6 (11.7% and 3.3%, respectively) than in the digestive tract of Control shrimp (2.3%).

By day 35, the genus *Celeribacter* and *Ruegeria* were the most abundant genera in the shrimp’s digestive tract, with a relative abundance of 17.3–31.1% and 27.2–39.9%, respectively. In the case of *Shimia*, significant differences were observed in the relative abundance in T3 (2.1%), T6 (3.5%) and Control shrimp (1.2%) (*p* < 0.001). As a lower represented genus (<1%), *Catenococcus* also showed significant differences among treatments (*p* < 0.05) and its abundance was higher in T3 shrimps than in Control and T6 shrimps.

## Discussion

The compensatory growth is a mechanism that allows organisms to improve nutrient utilization and growth when food is abundant along natural fluctuations, including aquatic environments (Fraser et al., 2007; Buckup et al., 2008). In the present study, shrimp under 3 and 6 days of 70% feed restriction showed compensatory growth with full recovery of growth after *ad libitum* feeding was restored. A complete recovery through compensatory growth was observed in several species of decapod crustaceans with different strategies of feed restriction. Wu & Dong (2002) observed that a complete compensation was achieved in juvenile Chinese shrimp *Fenneropenaues chinensis* (weight range 0.486–0.569 g) subjected to 6 days of fasting and 30 days of refeeding. In 40-day-old post-larvae *L. vannamei* (116 ± 4 mg, avg. wt.) fasted for 3 days, full compensatory growth was reached after 9 days of refeeding (Lin et al., 2008). Cyclical feeding strategies have also been successful in promoting full compensatory growth in *L. vannamei* fasted during 1 to 3 days and refed during 3 to 9 days (Zhu et al., 2016). Nevertheless, if the cyclical feeding protocol is severe, the compensatory response could not reach a full recovery of growth during the refeeding period, as observed in *L. vannamei* (0.46 ± 0.18 g, avg. wt.) exposed to repetitive cycles of 2 days of fasting and 1 day of feeding for 30 days, followed by a recovery re-feeding period of 28 days (Rocha et al., 2019). On the other hand, determination of the specific growth rate (SGR) provides a valuable tool to identify specific periods of high growth rates (Ricker, 1975). In the present study, shrimp showed a significantly higher SGR during the feeding recovery period, revealing a compensatory response, as observed in other studies conducted on the same species (Yu et al., 2008; Liu et al., 2022) and also in the Chinese shrimp *F. chinensis* (Zhang et al., 2010), in the Oriental river prawn *Macrobrachium nipponense* (Li et al., 2009) and in the crayfish *Cherax quaricarinatus* (Stumpf, Tropea & Greco, 2014; Stumpf & López Greco, 2015).

In some cases, a severe and long duration of the feed restriction could result in a partial growth compensation in shrimp, but leads to significant feed savings (Lara et al., 2017; Rocha et al., 2019; Yildirim & Aktaş, 2019). In the present work, treatments with feed restriction promoted between 5% and 12% feed saving as compared to Control shrimp. Furthermore, when natural food sources are available in the culture systems, as in biofloc systems, the feed saving could represent between 25% and 50% (Lara et al., 2017; Maciel, Francisco & Miranda-Filho, 2018; Rocha et al., 2019). Moreover, in many cases, and as determined in the present study, an improvement in feed conversion ratio (FCR) is observed in shrimp under compensatory growth (Stumpf & López Greco, 2015; Zhu et al., 2016; Rahman, Ghosh & Islam, 2020). A lower FCR implies a better nutrient utilization by shrimp and, therefore, a reduction in water pollution (Chaikaew et al., 2019).

Regarding the estimation of metabolic variables through stable isotope analysis, once the reference diet was provided in the experimental period, a fast influence of the isotopic signature on the shrimp muscle tissue was observed, showing a similar tendency of isotopic change among the three treatments. At the end of the experimental period, shrimp under all treatments approached isotopic equilibrium with values of δ^15^N = 14.31 ± 0.26‰, reflecting not only the fast growth, but also a high assimilation and utilization of nutrients supplied by the reference diet. In juvenile organisms, the isotopic equilibrium between animal tissue and diet can be attributed to the sum of growth and metabolic turnover (Gamboa-Delgado, 2022). Starvation can influence the capacity of shrimp to achieve isotopic equilibrium. The restriction of nutrients promotes high metabolic tissue turnover as a physiological response of the animal to supply essential nutrients. Nutrients are actually recycled at this stage and cause an enrichment of δ^15^N values in tissues (Bójorquez-Mascareño & Soto-Jiménez, 2016). Although the feed restrictions caused a significant reduction of the nitrogen turnover rates in muscle tissue, an isotopic equilibrium was still reached and it was mainly promoted by tissue accretion. Furthermore, shrimp during compensatory growth showed a lower contribution of the metabolic turnover (12–24%) to the overall isotopic change than that caused by the Control diet (39%), whereas nitrogen half-times (*t*_50_) were significantly higher (9.2–10 days) than those observed in the control group (8.3 days). The *t*_50_ values showed a tendency to increase in relation to the increase of shrimp growth, as observed in other studies conducted on the same species (Gamboa-Delgado et al., 2011, 2020). In this regard, shrimp under compensatory growth showed a higher contribution of growth (*k*) to the isotopic change (T6 = 88% and T3 = 76%) than animals belonging to the Control group (61%). The latter observations can be attributed to the higher growth rate elicited by the compensatory growth during this period. A higher SGR in juvenile shrimp reflects faster isotopic changes attributed to the dietary nitrogen contribution (and the intrinsic isotopic values) to tissue growth, therefore, isotopic equilibrium was reached primarily through shrimp somatic growth.

The increase on feed efficiency utilization is closely related to the regeneration of digestive tissues and the reactivation of enzymatic activities after the recovery period (Rocha et al., 2019; Zaefarian et al., 2020), which also promotes the restoration of energy reserves deployed during feed restriction (Wu & Dong, 2002; Rahman, Ghosh & Islam, 2020). In this regard, protein digestion and assimilation have a determinant role in shrimp growth, in which trypsin is one of the most important digestive proteases in shrimp (Lemos, Ezquerra & Garcia-Carreño, 2000). In the present work, shrimp under compensatory growth showed an increase of trypsin activity after 3 (T6) and 6 (T3) days of refeeding compared to the Control group, suggesting a higher protein utilization during the enhanced growth rate, as observed in *Macrobrachium rosenbergii* during compensatory growth (Rahman, Ghosh & Islam, 2020). Similarly, in the shrimp *F. chinensis*, an increase of trypsin activity was observed during the recovery feeding period after a period of fasting (Zhang et al., 2010). It has also been reported that *L. vannamei* reared under biofloc conditions, also showed an increase of trypsin during the refeeding period (Rocha et al., 2019).

In the present study, during the recovery period after 6 days (T3) shrimp showed higher amylase activity than those after 3 days (T6) of *ad libitum* feeding; nevertheless, no significant differences were shown compared to the Control treatment. Similar results were reported by Rocha et al. (2019) between shrimp after a feed restriction period and the Control group. Amylase is a digestive enzyme that facilitates the digestion of carbohydrates, which represents an economical source of energy for shrimp (Rosas et al., 2002). Sánchez-Paz et al. (2007) described that *L. vannamei* use glucose as a source of energy during short periods of starvation, and according to our findings, this physiological preference may change to protein and lipids at the beginning of the recovery period.

On experimental day 9, the lipase activity measured in shrimp under T6 treatment significantly increased when compared to the Control shrimp (3 days after feeding recovery), but it was no significantly different from the T3 treatment (6 days of feeding recovery). This result suggests a short-term increase of lipase activity during the refeeding period when the feed restriction is not prolonged. On the other hand shrimp in T3 and T6 showed hyperphagia after feed restriction, and therefore a higher intake of lipids, which play a determinant role for energy supply during compensatory growth (Maciel, Francisco & Miranda-Filho, 2018). Higher lipase activity is usually correlated to higher growth rates and final weight in *L. vannamei* shrimp (Gamboa-Delgado, Molina-Poveda & Cahu, 2003; Peixoto et al., 2018). In fish, elevated growth hormone during food deprivation promotes lipids mobilization for basal metabolism maintenance, meanwhile refeeding usually results in hyperphagia and hyperanabolism leading to a lipostatic return to energy homeostasis (Won & Borski, 2013). By day 14 of the experiment, shrimp showed similar digestive enzyme activities among treatments, suggesting a stabilization in the digestive functions as reported by other authors (Sacristán et al., 2016; Rocha et al., 2019; Shao et al., 2020). The selection of the feed restriction protocol is critical, since an excessively prolonged feed restriction may damage the digestive tract tissue, and affect the digestive enzymatic production after re-feeding, both conditions would limit the growth compensation (Abolfathi et al., 2012; Shaibani, Amiri & Khodabandeh, 2013).

Regarding the bacterial community in the shrimp gut, *Proteobacteria* was the dominant phylum, as previously reported in shrimp under different culture conditions (Sha et al., 2016; Pedrosa-Gerasmio et al., 2020; Holt et al., 2021). The second most abundant phylum was *Actinobacteria*, which is commonly reported with high abundance in the shrimp’s digestive tract (Pedrosa-Gerasmio et al., 2020). *Actinobacteria* is associated with healthy shrimp (Wu et al., 2016) and gut homeostasis (Binda et al., 2018). Nevertheless, no significant differences were found between treatments at this taxonomic level during the recovery period (day 14), nor at the end of the experiment (day 35). At the family taxonomic level, *Rhodobacteraceae* was the most represented group in all samples. Huang et al. (2016) and Xiong et al. (2017) have reported high abundance of *Rhodobacteraceae* in the intestine of *L. vannamei*, and its presence has been associated to the microbial core of healthy shrimp (Yao et al., 2018). On the other hand, during the compensatory period (day 14), a significantly higher relative abundance of *Vibrionaceae* was found in the Control shrimp compared to the rest of treatments; nevertheless, relative abundances were not significantly different at the end of the experiment. In shrimp *P. monodon* fed to satiety, the abundance of *Vibrio* tended to be higher than that observed in shrimp undergoing feed restriction (~60% of control diet) (Simon et al., 2020). Several bacterial species belonging to this group are pathogenic agents causing important economic losses in the farmed shrimp industry (de Souza Valente & Wan, 2021). Therefore, it can be argued that the feeding regimes T3 and T6 were indirectly beneficial for the health of shrimp during compensatory response.

At the genus taxonomic level, *Celeribacter* was one of the most abundant identified groups, showing a higher abundance in shrimps under the Control treatment (day 14). This genus has been described with high relative abundance (>10%) in the stomach of shrimp fed with a diet supplemented with 5-aminolevulinic acid (Pedrosa-Gerasmio et al., 2020). Furthermore, *Celeribacter* also show beta-glucosidase activity (Gao et al., 2019) that could improve carbohydrates digestion in shrimp. Additionally, *Catenococcus* and *Epibacterium* genera also showed significantly lower relative abundances in shrimp under compensatory growth. *Catenococcus* genus is related to the oxidation of sulfur compounds (Sorokin, Robertson & Kuenen, 1996) and has been reported as abundant in *Penaeus indicus* (Patil et al., 2021). *Epibacterium* is an abundant group found in macroalgal surfaces (Breider et al., 2019); however, the relation of both genera with shrimp is unknown. In the case of *Cohaesibacter*, it was only identified in the T6 treatment shrimps, and it has been described as a genus having high potential for bioremediation of aquaculture systems (Qu et al., 2011). Interestingly, treatments under compensatory growth showed an increase of ≥10% on the relative abundance of *Ruegeria*, and between 1% and 9.3% of *Shimia* compared to the Control group. These two genera have been described as beneficial bacteria that participate in phosphate and protein metabolism (Achbergerová & Nahálka, 2014; Yamaguchi et al., 2016; Duan et al., 2019). In the marine fish *Totoaba macdonaldi, Shimia* provides functional benefits to the intestine, improved nutrient absorption and growth (Barreto-Curiel et al., 2018), whereas *Ruegeria* exerts a probiotic function (Barreto-Curiel et al., 2018; Hasyimi, Widanarni & Yuhana, 2020).

Previous studies in crustacean and fish indicate that starvation can promote changes in the bacterial community structure with loss of bacterial diversity (Xia et al., 2014; Foysal et al., 2020; Sakyi et al., 2020). Crayfish *Procambarus clarkii* exposed to starvation during 3 days showed dysbiosis, with an increase in the number and abundance of species that limited ecological networks and reduced modularity; however, after 3 days of refeeding, the negative effects were reversed (Cai et al., 2022). In the present study, at day 14 during compensatory growth, bacterial diversity was similar among treatments and few differences were found within taxonomic levels. It is possible that a partial feed restriction applied in short periods (3 and 6 days) might have a lower impact on the bacterial community structure in the shrimp gut, or that the period (at day 14) was long enough to reestablish the potential change of bacterial diversity lost during feed restriction. Therefore, we suggest that a bacterial analysis over a short period after feeding recovery could provide complementary information to describe the bacterial successions occurring during compensatory growth.

## Conclusions

Shrimp under 3 and 6 days of feed restriction achieved full compensatory growth, leading to up to 12% feed savings after 35 days of experiment. During the feeding recovery period, the nitrogen metabolic turnover rate was lower in shrimp under compensatory growth than in the Control group shrimps, reflecting an increased dietary nitrogen utilization destined for growth. The digestive enzyme activities of trypsin and lipase increased during the initial days of compensatory growth to meet the requirement for amino acids and energy over the accelerated growth rate period. The bacterial community in shrimp gut was also modified during compensatory growth, with bacterial abundances pointing to potential benefits on nutrient metabolism and assimilation. When full compensatory growth was achieved at the end of the experimental period, the evaluated parameters showed similar results as those determined in the Control treatment, suggesting a normalization of metabolism and the physiological state. Further studies are required to determine if higher degrees of severity and duration of feed restriction, can differently affect the metabolism and microbial communities of shrimp during compensatory growth.

## Supplemental Information

10.7717/peerj.14747/supp-1 Supplemental Information 1 Raw measurements.

## Additional Information and Declarations

### DNA Deposition

The following information was supplied regarding the deposition of DNA sequences: Raw sequences are available at NCBI: PRJNA880846.

### Data Availability

The following information was supplied regarding data availability: The raw measurements are available as a Supplemental File.
